# Supplementary material for: Identification of T Cell Receptors Targeting a Neoantigen Derived from Recurrently Mutated FGFR3
Source: Cancers (Basel). 2023 Feb 6;15(4):1031. doi: 10.3390/cancers15041031 (PMC9953830; doi:10.3390/cancers15041031)
Supplement: Supplementary file 1 [file cancers-15-01031-s001.zip › Supplemetary Figures.pdf]

Index of Supplementary Data

Figure S1. Number of recurrent mutations and their patient coverage at different frequency thresholds in TCGA dataset.

Figure S2. *In-vitro* screening of shared neoantigen-reactive $CD8^+$ T cells. Figure S3. Transduction efficiency of generated TCR-engineered T cells.

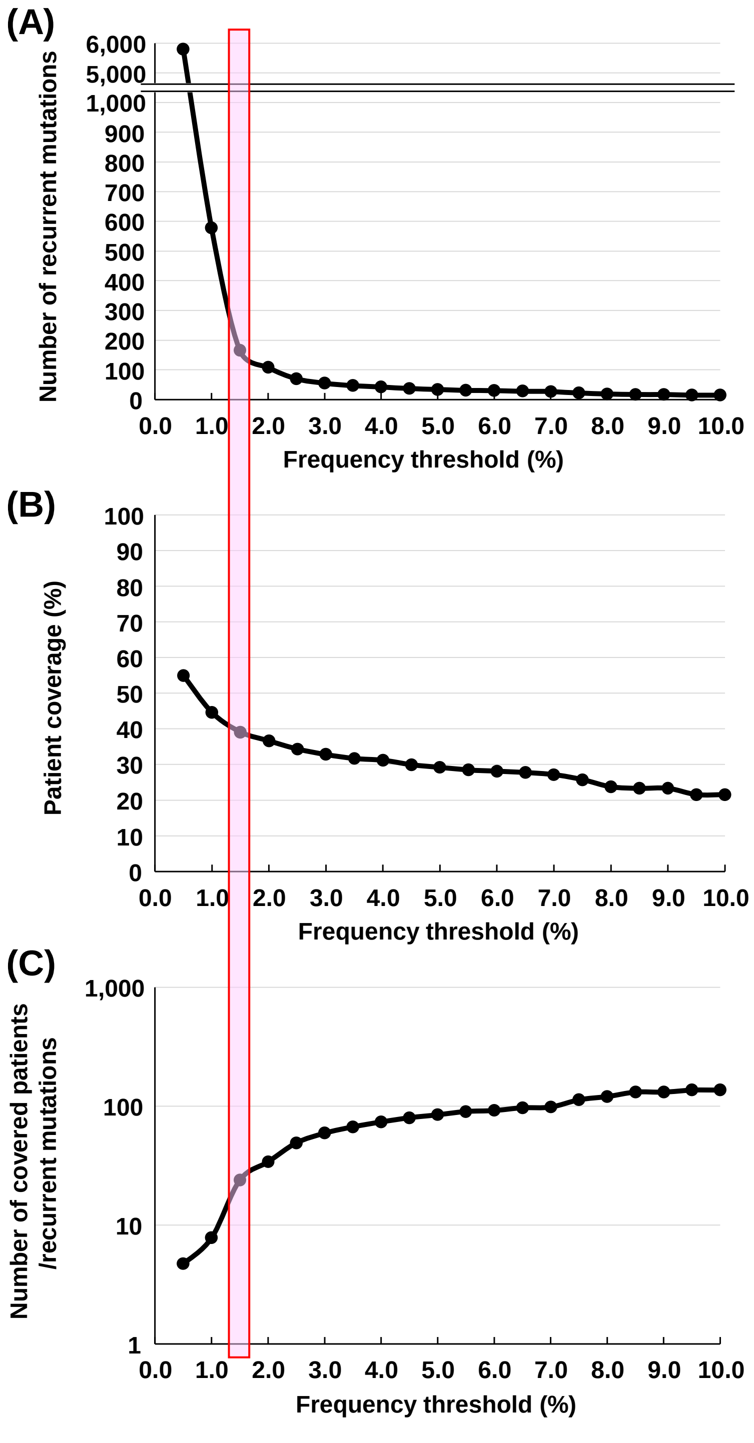

Figure S1. Number of recurrent mutations and their patient coverage at different frequency thresholds in TCGA dataset.

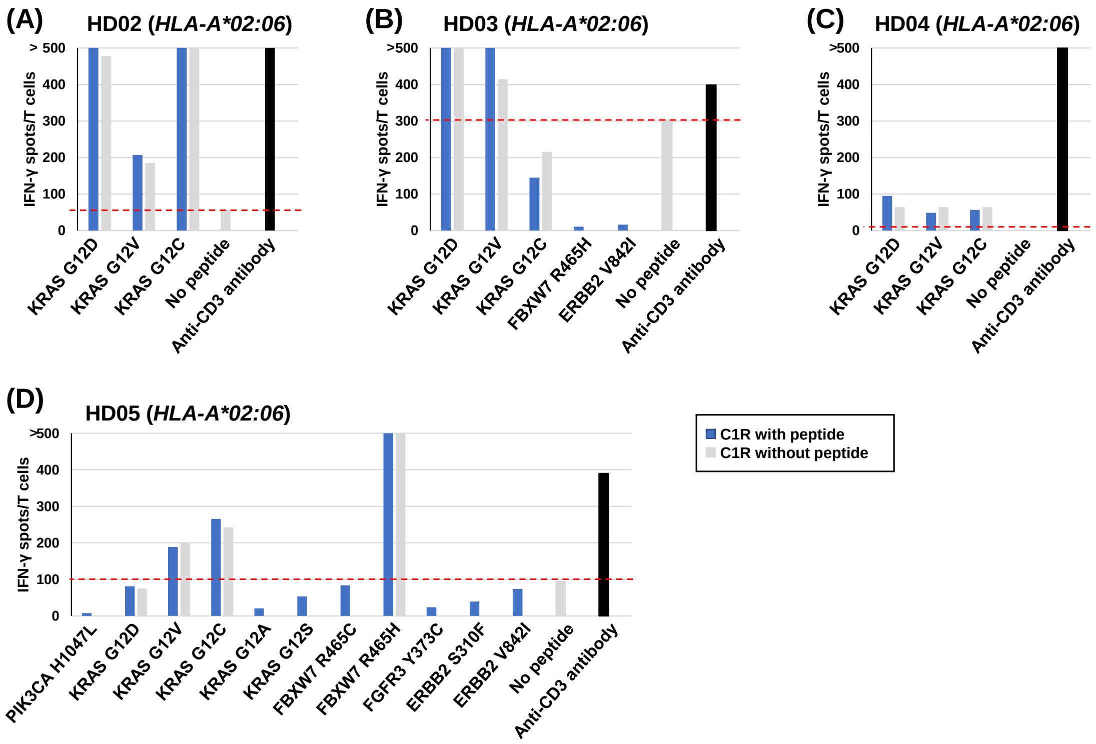

Figure S2. *In-vitro* screening of shared neoantigen-reactive CD8⁺ T cells. IFN-γ ELISPOT screening of CD8⁺ T cells for predicted shared neoantigen peptides using 4 healthy donors with *HLA-A*02:06* (HD02 – HD05, **A** to **D**) with different HLAs. Antigen-stimulated CD8⁺ T cells were re-stimulated with C1R-A0206 cells pulsed with or without shared neoantigen peptides. The data are represented as the means in duplicate experiment. Red dash lines represent background IFN-γ ELISPOTs in CD8⁺ T cells stimulated without peptide pulse autologous DCs.

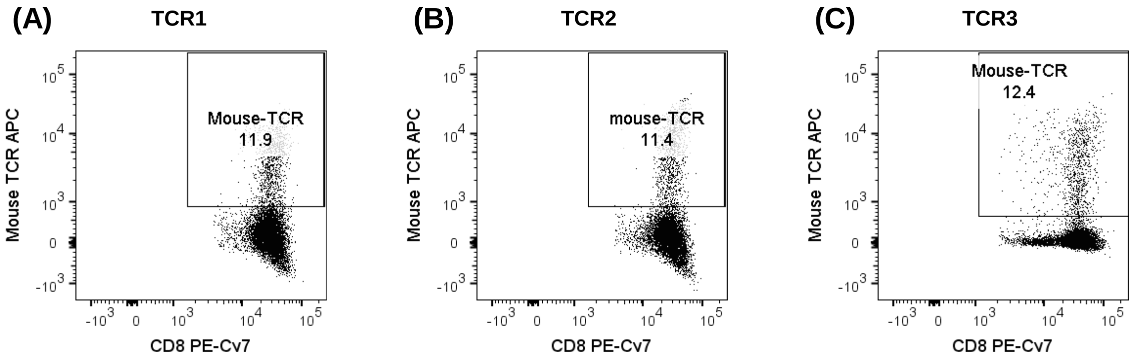

Figure S3. Transduction efficiency of generated TCR-engineered T cells. The expressions of engineered TCRs were confirmed using anti-mouse TCRβ antibody.
